# Association between urban density, built environment quality, and health: a multi-source study in Doha, Qatar

**DOI:** 10.3389/fpubh.2026.1865979

**Published:** 2026-06-18

**Authors:** Hameda Janahi, Manal Shghaiwi, Ahmed Alnuaimi, Reem Awwaad

**Affiliations:** 1 Department of Architecture and Urban Planning, College of Engineering, Qatar University, Doha, Qatar; 2 Clinical Affairs—Clinical Research, Primary Health Care Corporation, Doha, Qatar

**Keywords:** built environment quality, Doha, non-communicable diseases (NCD), residential density, urban health, walkability

## Abstract

**Introduction:**

Rapid urbanization in hot-arid, car-dependent cities challenges the widely accepted assumption that urban density inherently promotes public health. While higher residential density is often associated with increased physical activity and improved health outcomes in Western contexts, its effects in cities such as Doha, Qatar, remain insufficiently understood. This study examines the relationship between residential density, environmental quality, and health outcomes in Doha’s rapidly developing urban environment.

**Methods:**

A cross-sectional study was conducted using a mixed-methods approach that triangulated self-reported health and quality-of-life data collected through the SF-36 survey (N = 301), clinical records on non-communicable disease (NCD) prevalence obtained from Primary Health Care Corporations, and geospatial analyses of land use patterns and urban infrastructure across three residential zones in Doha. Associations between residential density, environmental conditions, and health indicators were examined.

**Results:**

Residential density was found to be differentially associated with health outcomes. Higher-density environments were associated with improved mental health and functional well-being; however, they were also associated with higher prevalence of non-communicable diseases and increased body mass index. These patterns appear to be influenced by contextual factors including inadequate pedestrian infrastructure, exposure to extreme heat, and limited climate-responsive urban design. The findings were particularly evident among vulnerable population groups engaged in necessity-driven walking.

**Discussion and conclusions:**

The results demonstrate that density alone is not a sufficient predictor of positive health outcomes in hot-arid urban environments. Environmental quality and the provision of climate-responsive infrastructure appear to play a more important role in shaping public health outcomes than density itself. The study highlights the need for urban planning and public health policies that move beyond densification strategies and prioritize walkability, thermal comfort, and high-quality urban environments to support NCD prevention and healthier lifestyles in rapidly developing cities.

## 1 Introduction

The global rise of non-communicable diseases (NCDs) is closely associated with the social and physical environments of cities, making urban design an important context for understanding public health patterns. The spatial configuration of cities, through factors such as residential density, land-use mix, and mobility networks, has been linked to daily behaviors and exposure patterns associated with chronic disease risk (1–4). However, these associations may operate differently in hot-arid, car-dependent contexts such as those of the Gulf Cooperation Council (GCC) region, where conventional assumptions about links between density and health are complicated by climatic constraints and car-oriented urban form.

In such environments, extreme heat and humidity are associated with substantial physiological constraints on outdoor activity for much of the year, which may reduce the walkability benefits often described in temperate climates (5). A deeply ingrained car culture, together with urban designs that prioritize vehicular movement over pedestrian infrastructure, is also associated with fewer opportunities for active transport regardless of residential density (6). This raises a critical question: are the established associations between urban form and health consistent across contexts, or do they vary with socio-cultural and climatic conditions that remain underexplored in mainstream research?

Doha, the capital city of Qatar, offers a compelling case through which to examine these dynamics. Fueled by hydrocarbon wealth, Doha has undergone rapid transformation over the past few decades, evolving from a modest coastal settlement into a rapidly expanding global metropolis. Today, its urban fabric includes modern high-rise developments, sprawling low-density villa compounds, and an extensive highway-oriented transportation network. This rapid, car-centric urbanization has been associated with major lifestyle changes and growing public health concerns, particularly the rising prevalence of NCDs (7–10).

While prior studies have documented the rising burden of NCDs in Qatar (8, 11) and others have examined selected aspects of its built environment, empirical evidence linking these two domains remains scarce. Specifically, little is known about how residential density and environmental quality are associated with NCD prevalence and Health-Related Quality of Life in hot-arid urban settings. This urban health study addresses the following questions:

- How is residential density associated with physical, mental, and social health outcomes in Doha?
- How is built environment quality associated with this relationship?
- What roles do individual and perceptual factors play in these associations?

Accordingly, the study aims to: (a) quantify associations between residential density and health outcomes across three residential zones in Doha; (b) evaluate how built environment quality and mobility behavior are associated with these patterns; and (c) contribute to a context-sensitive understanding of urban health in hot-arid, rapidly developing cities.

## 2 Literature review

A robust body of urban health research highlights the built environment’s role in shaping physical, mental, and social well-being (12, 13). Within this field, the concept of residential density is a central, yet highly contested, topic. This review synthesizes current knowledge on the density-health relationship. First it outlines the conventional paradigm, then examining the contextual factors that challenge it. Second, it establishes the methodological framework and specific research gap addressed in this paper.

Theoretically, the paper integrates concepts from environmental epidemiology, sustainable urban design, and the Social Determinants of Health (SDH) to develop a holistic understanding of well-being in the built environment (14). It reconceptualizes density not as a static indicator but as a relational construct whose impacts depend on spatial quality, accessibility, and climatic adaptation. The SDH model provides a robust theoretical basis for understanding how health outcomes are shaped not only by biological factors but also by the broader social, economic, and environmental conditions in which people live. It distinguishes between structural determinants (such as governance, income, education, and urban policy) and intermediary determinants, which include material conditions, psychosocial environments, behaviors, and access to health-promoting resources (15, 16). Together, these determinants form an interrelated system that produces and sustains health inequities across populations.

### 2.1 Residential density and urban health

International scholarship generally identifies a positive association between residential density and urban health. Dense urban forms promote proximity to destinations, encourage active transport such as walking and cycling, and reduce car dependency (2, 17, 18). Higher physical activity is associated with lower rates of obesity and related NCDs, including type 2 diabetes and cardiovascular disease (1, 19, 20).

However, this relationship is not universal. High or poorly planned density can also produce adverse health effects, such as crowding-related stress and lower mental well-being (3, 4, 21), which are collectively associated with poorer health outcomes (22–26).

Understanding the contextual factors mediating the density–health link is therefore essential. Environmental quality, climate, and socio-cultural conditions are strongly associated with this relationship. In hot-arid, car-oriented contexts such as Doha, these mediators are critical, making Western-derived urban health models insufficient and underscoring the need for context-sensitive approaches.

This is where the SDH model becomes critical. The conceptual model of the SDH was first articulated by Dahlgren and Whitehead (27) and later advanced by the WHO’s Commission on Social Determinants of Health in 2008 (14). It offers a multi-level understanding of how health is shaped by the social, economic, and environmental conditions in which people live (28). It distinguishes between structural determinants (such as socioeconomic and political mechanisms, governance, education, income) and intermediary determinants (including material conditions, psychosocial factors, behaviors, and healthcare access) (15, 28, 29). These determinants operate across scales, producing health inequities within and between communities. Importantly, the SDH moves beyond behavioral or biomedical explanations by emphasizing that individual choices and health outcomes are embedded within—and constrained by—the built, social, and environmental systems that define everyday life (16, 30, 31).

Critiques of the SDH model note its narrow focus on income or education, often neglecting spatial and perceptual factors. Recent studies advocate integrating built environment and climatic variables as mediating layers within the SDH model (32). Health outcomes emerge from multi-scalar, interacting systems rather than isolated indicators like density. Thus, urban form acts as both a structural and intermediary determinant. In rapidly urbanizing, hot-arid, and car-dependent contexts such as Doha, adapting SDH to include spatial and climatic dimensions is crucial for understanding how form and social structures shape health.

The GCC region exemplifies such contexts, where extreme climate and mobility patterns reshape how density is associated with health. The assumptions underpinning the conventional density-health paradigm are most severely tested in hot-arid, car-dependent cities like those of the GCC region. In these contexts, a confluence of environmental and socio-cultural factors acts as a powerful set of mediating variables that often neutralize or even reverse the theorized health benefits of density.

Qatar reports some of the highest rates of NCDs in the region, with an estimated diabetes prevalence of 15.5% in 2021 and obesity affecting more than 40% of the adult population (7). These diseases are among the leading causes of death in the country and are associated with high levels of physical inactivity (8, 9). The urban fabric of Doha reveals striking spatial contrasts, ranging from hyper-dense developments like Msheireb (26,111 people/km^2^) to low-density suburban areas like Al Thumama (3,883 people/km^2^) (33). Despite Qatar’s National Health Strategy (NHS) and the Third National Development Strategy (NDS3) prioritizing health-conscious urban planning, localized empirical research linking built environment indicators to health outcomes remains limited (9). Applying an SDH lens to this context allows for these variables (climatic stress, car dependency, and infrastructural inequality) to be understood not as anomalies but as integral determinants of health within the urban system.

These national health and spatial realities highlight the need to examine density through a localized lens. A key challenge is Qatar’s extreme climate, which poses a major physiological barrier to physical activity. For much of the year, high heat and humidity make outdoor spaces uncomfortable and even unsafe, undermining the benefits of walkable urban forms (1–4). Empirical evidence supports this: in Abu Dhabi, pedestrian-friendly areas still saw reduced outdoor activity due to thermal discomfort (34). Similar patterns appear in other hot, car-dependent cities, where extreme heat often negates the intended benefits of compact, walkable urban design (35).

Beyond climate and infrastructure, the relationship between urban form and health is also shaped by individual and social factors (6). Demographics and perceptions strongly associated with health behaviors. Age and gender are key determinants, with older adults facing higher risks for NCDs such as hypertension (8, 11). Cultural norms—particularly those related to gender—further associated with mobility and access to public space. In Doha, where many adults are aged 51 and above (33), these demographic and cultural dynamics act as critical confounders that must be addressed when analyzing health outcomes at the neighborhood scale.

Residents’ subjective perceptions of their environment are powerful determinants of physical activity and mental health. These perceptions extend beyond walkability, encompassing sensory and psychological cues (36). Visual and aesthetic qualities—such as public art, building upkeep, and perceived order—shape how residents evaluate their surroundings (37). Perceptions of safety, from both traffic and crime, and the presence of pleasant, well-maintained green spaces further associated with neighborhood satisfaction and outdoor activity (24). While objectively walkable environments enhance satisfaction and Health-Related Quality of Life (HRQoL) in Doha, potential gaps between objective quality and subjective perception warrant examination (1, 38). Understanding how these perceptions interact with density and behavior is central to this study’s holistic analysis.

### 2.2 Integrated methods for assessing urban health and environmental quality

A growing body of research investigating the nexus between urban characteristics and health has adopted diverse methodological approaches, ranging from geospatial epidemiology to psychometric surveys. Central to such work is the measurement of health outcomes in a way that captures both objective and subjective dimensions of wellbeing. Among the most widely validated tools for this purpose is the Short Form Health Survey (SF-36), which offers a comprehensive measure of HRQoL across multiple domains. The SF-36 assesses eight core health dimensions—physical functioning, role-physical, bodily pain, general health, vitality, social functioning, role-emotional, and mental health—generating two composite indices: the Physical Component Summary (PCS) and the Mental Component Summary (MCS) (39).

The SF-36 has been extensively validated in both Western and non-Western populations and has demonstrated strong internal consistency and construct validity across different cultural contexts, including Arabic-speaking countries (40, 41). In urban health studies, the instrument has gained increasing relevance for its ability to detect nuanced variations in perceived wellbeing that may be influenced by environmental quality, accessibility, and exposure to climate stressors (12, 42).

Recent studies have utilized the SF-36 within similar urban research paradigms. For instance, a study in Qingdao, China, used the SF-36 to investigate how built environment factors such as greenery, pollution, and walkability correlated with chronic disease prevalence at the neighborhood level (42). Similarly, a recent review study has employed SF-36 metrics to assess child wellbeing in high-density environments, revealing that infrastructural quality and social equity often mediate health outcomes more strongly than density per se (12).

Overall, this paper builds upon a well-established methodological tradition, adapting it to Doha’s unique climatic, cultural, and infrastructural context. By integrating the SF-36 survey with clinical and geospatial datasets, it provides a robust, context-sensitive foundation for examining the relationship between residential density and health. The literature emphasizes that this relationship is complex and highly context-dependent: while higher density can enhance health through improved accessibility and active mobility, outcomes are strongly mediated by environmental quality, climate, socio-cultural dynamics, and individual perceptions. In hot-arid, car-oriented settings, these mediators are particularly influential, necessitating an integrative framework that reconceptualizes density as a relational construct shaped by spatial quality, accessibility, and climatic adaptation. Integrating the SDH model provides this paper with a rigorous theoretical grounding. It advances an evidence-based, context-sensitive model that links spatial form to population health, contributing to both global urban health scholarship and Qatar’s policy agenda for sustainable, people-centered development.

## 3 Methodology

This paper adopts an exploratory cross-sectional triangulation design, combining spatial metrics with self-reported health outcomes in an ecological-comparative framework. All findings reported in this paper should be interpreted as associations between built environment characteristics and health outcomes; no causal relationships are implied, and the possibility of reverse causality cannot be ruled out given the cross-sectional design. This multi-source quantitative approach is guided explicitly by the Social Determinants of Health (SDH) model, developed by the World Health Organization and further elaborated by Solar and Irwin (14, 28). The paper positions the SDH model as its core theoretical foundation—linking structural, environmental, behavioral, and perceptual determinants of health within the specific spatial and climatic realities of Doha. This approach bridges a critical gap between urban planning and public health scholarship in the Middle East, offering insights with both academic and policy relevance.

Building upon this foundation, the paper introduces a context-sensitive adaptation of the SDH model to the urban and climatic realities of Doha. While the traditional SDH model emphasizes socioeconomic and political drivers of health, this adaptation extends the model to include spatial and environmental determinants—particularly those related to residential density and built environment quality. This approach recognizes that in hot-arid, car-dependent cities, urban form plays a crucial mediating role between structural and behavioral determinants of health. Residential density, for instance, is associated with the accessibility of services, exposure to environmental stressors, and opportunities for physical activity, all of which are integral intermediary determinants in the SDH model. The built environment—its quality, permeability, and comfort—thus becomes a structural and behavioral conduit through which social and climatic inequities are expressed in health outcomes (32).

The methodological rationale, therefore, recognizes that urban form is associated with health through multiple, interacting SDH-informed pathways: structural (such as land use, accessibility, and infrastructure), environmental (including air quality, microclimate, and shade provision), behavioral (such as physical activity levels, transport choice, and social interaction), and perceptual/psychosocial (including safety, satisfaction, and perceived neighborhood quality). Understanding these interactions is critical to capturing the multidimensional nature of wellbeing in Doha’s rapidly urbanizing, high-heat context.

Among the various quantitative approaches, the SF-36 survey was employed in this paper due to its comprehensive coverage of physical, social, and mental health domains, strong psychometric properties, and proven cultural adaptability in Arabic-speaking populations (39). The Arabic SF-36 version has demonstrated acceptable internal consistency across validation studies in Arabic-speaking and GCC populations, with Cronbach’s alpha values ranging from 0.72 to 0.90 across subscales (39).

All findings are interpreted as associations between residential density, built-environment quality, and self-reported or clinical health indicators. Given the cross-sectional, exploratory, case-comparative design of the study, no causal inferences are drawn, and the direction of any observed relationship cannot be established from the present data.

While qualitative studies have enriched urban health research by providing contextual insights, a persistent limitation has been the reliance on single-source self-reported data (43). To align with the SDH’s multi-layered structure, the paper adopts a multi-source quantitative triangulation approach, integrating:

- Survey data on self-reported health and neighborhood environmental perceptions (via SF-36 survey);
- Objective, anonymized clinical data on non-communicable diseases (NCDs) obtained from Primary Health Care Centers (PHCC); and
- Geospatial and observational data from on-site assessments of built environment quality, density typologies, and accessibility.

**Figure 1:**
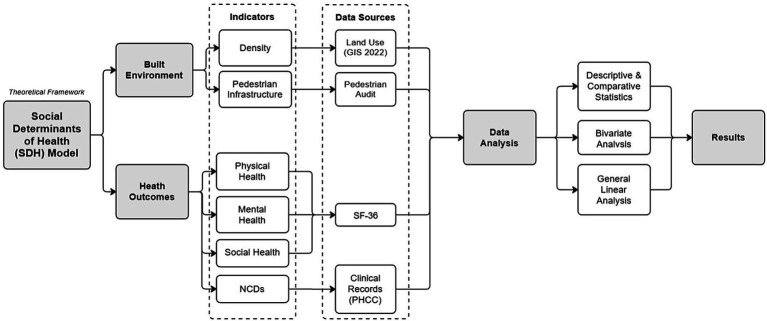
The research design based on the Social Determinants of Health (SDH) model.

A schematic overview of the research design is presented in Figure 1. It illustrates the integration of built environment and health outcome variables through defined indicators, data sources, and analytical methods. The SDH model guides the relationship between environmental factors (density and pedestrian infrastructure) and health outcomes (physical, mental, social health, and NCDs), analyzed using descriptive, comparative, correlation, chi-square, internal consistency (Cronbach’s *α*; (39, 44)), and adjusted general linear model (GLM) statistical methods—controlling for zone, age, gender, nationality, and employment status as socio-demographic covariates to produce the study results.

### 3.1 Case study zones

**Figure 2:**
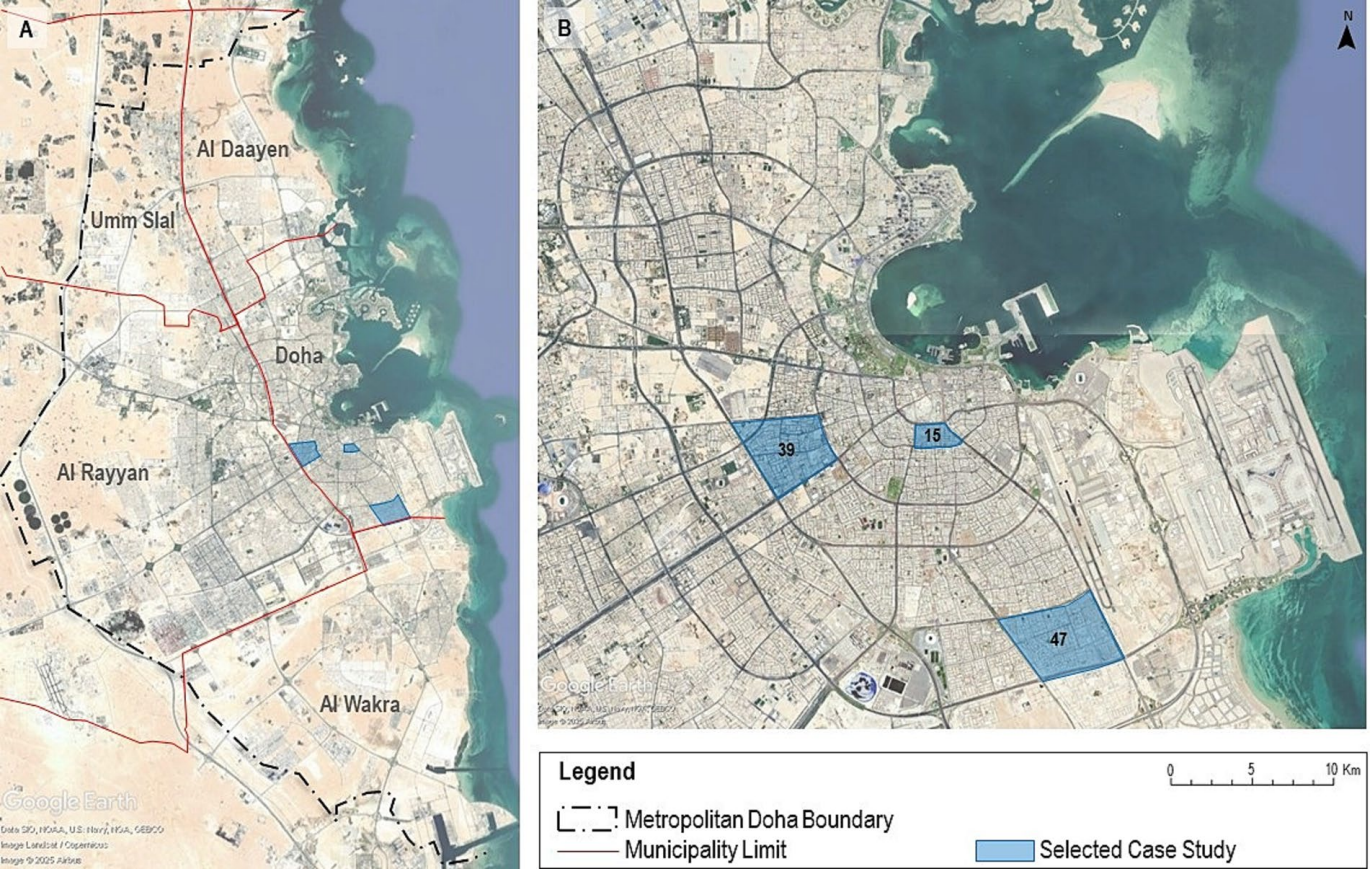
**(A)** Location map of the case study zones at the scale of Metropolitan Doha, illustrating the study’s overall spatial context; **(B)** Detailed view of the selected zones—Zone 15 (Ad Dawhah Al Jadidah), Zone 39 (Fereej Al Nasr), and Zone 47 (Al Thumama)—representing distinct population densities across the city (Image © Google, Data SIO, NOAA, U.S. Navy, NGA, GEBCO, Landsat/Copernicus, Image © 2025 Airbus).

To capture a representative range of Doha’s diverse urban typologies, a stratified case study selection was employed. The primary selection criterion was residential density, with zones categorized based on official population density thresholds into High (>8,000 residents/km^2^), Medium (5,000–8,000 residents/km^2^) and Low (<5,000 residents/km^2^) categories (33). From these strata, one zone is selected for each category: Zone 15 (Ad Dawhah Al Jadidah), Zone 39 (Fereej Al Nasr), and Zone 47 (Al Thumama) (Figure 2).

A secondary set of criteria was applied to strengthen the robustness and validity of the analysis. The selected zones represent diverse geographic locations within Doha and exhibit varied land use profiles. This ensures that the study captures different types of urban form beyond a mere density metric (Table 1).

**Table 1:** Overview of the case study zones, detailing their respective areas, population density, and land use composition (building counts).

Zone number	15	39	47
Zone name	Ad Dawhah Al Jadidah	Fereej Al Nasr	Al Thumama
Area (km^2^)	0.5	2.6	3.1
Population density (people/km^2^)	31,840	8,830	3,883
Density category	High density	Medium density	Low density
Agricultural and green areas	3	6	19
Educational	8	22	29
Governmental	7	23	28
Mixed use	11	132	93
Open space & recreation	8	11	27
Religious	4	22	35
Special use	18	189	65
Sports	3	5	15
Tourism	1	5	5

### 3.2 Data sources

Data for this study were derived from three sources to enable comprehensive triangulation: (1) questionnaire survey (to collect self-reported health data and perceptual data), (2) clinical dataset from PHCC (to collect health data); and (3) spatial analysis and infrastructure assessment (to collect land use and GIS data).

#### 3.2.1 Questionnaire survey

A structured, self-administered questionnaire was designed to capture data across four domains: (1) Health-Related Quality of Life (HRQoL), measured using the SF-36 survey and its validated Arabic version. The eight SF-36 domain scores were used to compute the Physical Component Summary (PCS) and Mental Component Summary (MCS) scores by applying factor-score coefficients derived from the U.S. general population normative dataset, following the standard scoring procedures described by (39). (2) Health status and behaviors, including self-reported body mass index (BMI) and lifestyle patterns. (3) Neighborhood perceptions, assessed through Likert-scale items capturing residents’ views of their local environment; and (4) socio-demographic characteristics, covering standard background variables. The survey was conducted in accordance with the Declaration of Helsinki, and ethical approval was obtained from the Institutional Review Board of Qatar University (Approval No. QU-IRB 305/2024-EM).

Statistical analyses were performed using the software IBM SPSS Statistics version 28. Descriptive statistics summarized respondent characteristics by zone. Internal consistency of the SF-36 subscales in the present sample was assessed using Cronbach’s *α*, following the standard reverse-coding procedures specified by (39) prior to subscale computation. Bivariate associations between residential density and health outcomes were examined using Pearson and Spearman correlations and chi-square tests, with effect sizes reported where appropriate.

To address potential socio-demographic confounding at the respondent level, two adjusted general linear models were fitted with the PHS and MHS T-scores as dependent variables. Each model included zone, age group, gender, nationality, and employment status as fixed factors. Employment status was included as a proxy for socio-economic position, as direct income data were not collected in the survey. Pairwise zone comparisons were conducted on estimated marginal means with Bonferroni adjustment for multiple comparisons. Effect sizes are reported as partial *η*^2^ (44). Statistical significance was set at *p* < 0.05. Spatial-statistical methods (e.g., Moran’s *I*, geographically weighted regression) were not applied, as the three zones constitute too few spatial units to support meaningful spatial-autocorrelation testing; the spatial component of the study is therefore reported descriptively as land use and pedestrian-infrastructure characterization.

To mitigate the limitations of self-reported data, the study triangulated SF-36 findings with objective clinical records, biometric indicators, and geospatial data. This multi-source design, incorporating PHCC clinical records, land use analysis, and pedestrian infrastructure audits, strengthens internal validity by reducing single-source bias and enabling cross-verification (21, 31).

A target sample size of approximately 300 participants was set, with 100 respondents intended for each zone. A total of 301 responses were collected across the three zones: *n* = [107] in Zone 15 (high density), *n* = [103] in Zone 39 (medium density), and *n* = [91] in Zone 47 (low density). Participants were recruited between February and May 2025 using a two-stage hybrid sampling strategy. First, initial intercept sampling was conducted by research assistants who approached eligible residents in high-traffic public spaces and amenities within each zone, including parks, local shops, and transit points. These initial participants served as seeds for the snowball phase. Second, each participant who completed the digital survey was asked to share the survey link with family members and neighbors residing in the same zone, thereby extending recruitment through localized social networks. Eligible participants were adults who had lived in the selected zone for at least 5 years.

The socio-demographic composition of the sample (Table 2) is disaggregated by zone and includes gender, age group, nationality, length of residence, employment status, and marital status. This distribution provides the necessary contextual baseline for comparing population characteristics across density gradients and for interpreting subsequent variations in HRQoL, behavioral patterns, and neighborhood perceptions.

**Table 2:** Descriptive demographic characteristics of the questionnaire survey respondents by zone.

Zone number		15	39	47
Zone name		Ad Dawhah Al Jadidah	Fereej Al Nasr	Al Thumama
Gender	Male	81	47	38
	Female	26	56	53
Age group (years)	18–30	38	33	36
	31–50	61	53	40
	51–65	8	17	18
	+66	-	-	-
Nationality	Citizen	8	13	49
	Non-citizen	99	89	45
Length of residence (years)	Less than 5	30	10	15
	6–9	37	16	22
	More than 10	40	77	57
Employment status	Employed	83	71	39
	Not employed	5	11	22
	Retired	5	5	6
	Student	14	12	24
Marital status	Married	33	56	53
	Single	43	33	27
	Prefer not to say	25	12	6
	Widowed	3	-	2
	Divorced	3	2	6

#### 3.2.2 Clinical datasets from PHCCs

Clinical datasets were procured from the PHCC to provide information of aggregated, zone-level prevalence rates for diagnosed NCDs among adults 18 years and older for the year 2024. PHCC is Qatar’s largest publicly funded primary care provider in Qatar. It operates health care centers distributed all over Qatar. PHCC uses a well-articulated electronic health record (EHR) system. Data for the purposes of this study was extracted from PHCC’s EHR for residents of the three selected zones. A total of 25,023 adults qualified for this anonymized data extraction during the year 2024. Reviewing SNOMED codes of the extracted EHR a confirmed clinical diagnosis for one of four selected NCDs (Diabetes, Blood Pressure, Heart Disease, and Chronic Lung) was verified. In addition, the height and weight were also extracted when available to label the individual as overweight/obese if the BMI is 25 kg/m^2^ and more, or obese if its ≥ 30 kg/m^2^. The study presented minimal risk of harm to human participants. The data extracted from health records were anonymized ensuring that none of the participants’ personal information was revealed to the research team. The study was conducted with integrity adhering to generally accepted ethical principles and was approved by the PHCC’s Institutional Review Board (IRB reference number BUHOOTH-D-25-00056).

#### 3.2.3 Built environment and geospatial data

**Figure 3:**
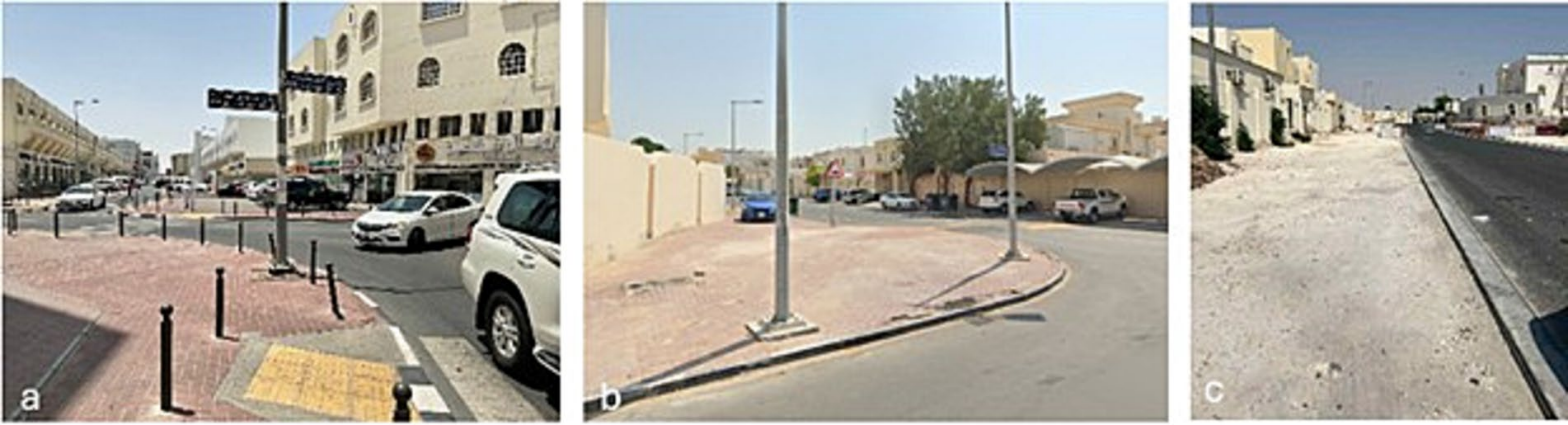
Examples of sidewalk quality classification **(a)** good condition; **(b)** medium condition; and **(c)** poor condition.

To objectively characterize the physical context of the study areas, a dual methodological approach was adopted, integrating geospatial land use data with field-based pedestrian infrastructure assessments. Quantitative land use information for the three study zones was obtained from the Centre for GIS (CGIS) (45), providing detailed classifications and parcel counts within each zone’s boundary to facilitate an objective evaluation of urban form, density, and amenity distribution (45). Complementing this macro-scale analysis, a systematic on-site assessment of pedestrian infrastructure was undertaken to document micro-scale variations in built environment quality. Sidewalks and pathways were evaluated through direct observation, location-tagged photographs, and annotated maps, ensuring spatial accuracy and methodological consistency across zones. Field assessments were conducted by undergraduate architecture students trained in the observation protocol between October 2024 and January 2025. Sidewalk quality was classified independently using a standardized rubric and then cross-checked for consistency across zones. The rubric grouped sidewalks into three categories: Good (continuous, unobstructed, level, well-maintained, and safe for all users), Medium (minor surface defects, limited unevenness, or occasional obstructions not impeding passage), and Poor (discontinuous, severely damaged, obstructed by vehicles or debris, overgrown, or otherwise unsafe and functionally unusable). This combined geospatial and fieldwork approach provided a comprehensive, empirically grounded characterization of the urban physical environment across the study zones (Figure 3).

## 4 Findings

### 4.1 Self-reported health outcomes verses built environment quality

The study sample (*N* = 301) was predominantly male (55%), with notable demographic variation across the three zones. Zone 47, the low-density area, had a proportionally older age profile, particularly in the 51–65-year group, than Zone 15, the high-density area. This age distribution may have influenced the observed health patterns across zones and should be considered when interpreting the associations between residential density and health outcomes. These results indicate a complex association between residential density and public health, with demographic composition likely contributing to the observed variation.

The socio-demographic profile of the sample reveals several structural imbalances across zones that may influence the interpretation of density–health relationships. Zone 15 (high density) is characterized by a younger, predominantly male, and overwhelmingly non-citizen population, whereas Zone 47 (low density) is comparatively more gender-balanced with a substantially higher proportion of citizens. Zone 39 (medium density) exhibits a more stable residential profile, with a larger share of long-term residents (>10 years) and a relatively older age structure, particularly in the 51–65 cohort. Although older adults representation (+66) is minimal across all zones, the relative ageing in Zones 39 and 47 compared to the younger profile of Zone 15 suggests potential confounding effects, particularly for health outcomes and Health-Related Quality of Life (HRQoL) measures. These disparities—especially in gender composition, nationality, and residency duration—are not evenly distributed across density gradients and may partially mediate or obscure observed associations between residential density and both perceived and clinical health indicators.

**Figure 4:**
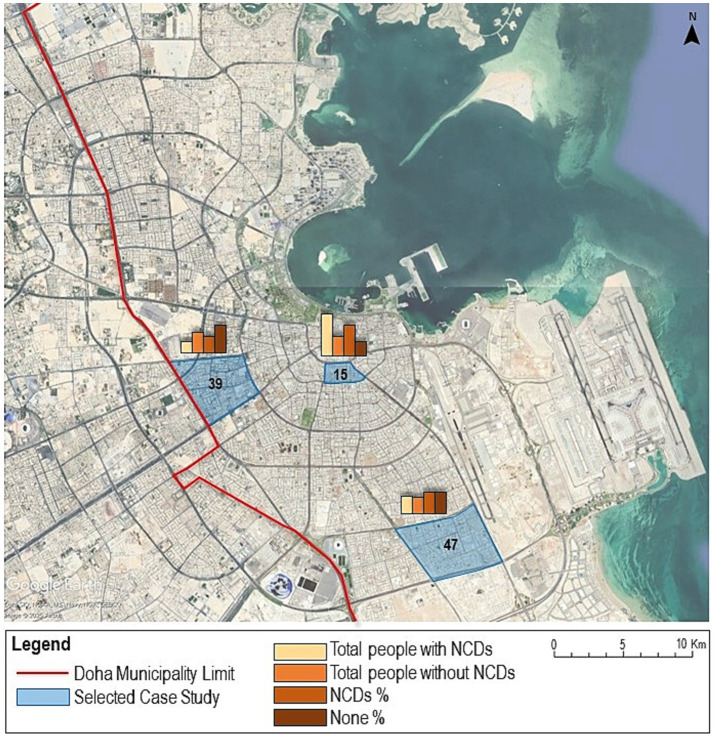
Distribution of NCD burden (% with NCDs vs. % None) across the studied zones in Doha (Image © Google, Data SIO, NOAA, U.S. Navy, NGA, GEBCO, Landsat/Copernicus, Image © 2025 Airbus).

**Figure 5:**
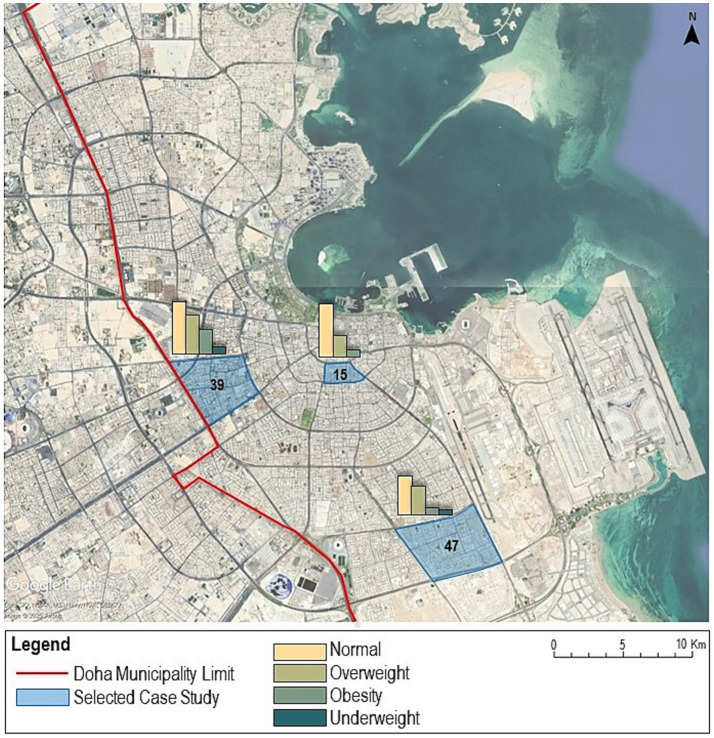
Distribution of Body Mass Index (BMI) categories across the studied zones in Doha (Image © Google, Data SIO, NOAA, U.S. Navy, NGA, GEBCO, Landsat/Copernicus, Image © 2025 Airbus).

Higher residential density in Doha was significantly associated with poorer physical health indicators. Bivariate Pearson correlations showed that as density increased, so did the prevalence of self-reported diabetes, high blood pressure, and chronic lung disease (all *p* < 0.026). Chi-square tests confirmed these findings, revealing that 67.9% of residents in high-density zones reported at least one NCD, compared to 51.3% in low-density and only 38.57% in medium-density zones (Figure 4). This pattern was mirrored in body mass index (BMI), with a higher proportion of obesity observed in the central, high-density zones (Figure 5).

The correlation analysis revealed a set of statistically significant relationships between residential density and several dimensions of the SF-36 HRQoL measure (Table 3). Results show that higher residential density was positively associated with several domains of health and wellbeing, particularly physical functioning (*ρ* = 0.183, *p* = 0.001), role physical (ρ = 0.170, *p* = 0.003), role emotional (ρ = 0.156, *p* = 0.007), and mental health (*ρ* = 0.174, *p* = 0.002). These associations suggest that residents in denser neighborhoods tend to report better perceived functioning and mental resilience.

**Table 3:** Spearman’s rho correlations between residential density and health outcomes (*N* = 301).

Outcome Category	Variable	Spearman’s rho (*r*)	Sig. (2-tailed)	Significance
HRQoL—individual scales (0–100)	General health (GH)	−0.036	0.530	n.s.
	Physical functioning (PF)	0.183	0.001	*p* < 0.01
	Role-physical (RP)	0.170	0.003	*p* < 0.01
	Role-emotional (RE)	0.156	0.007	*p* < 0.01
	Social functioning (SF)	0.081	0.160	n.s.
	Bodily pain (BP)	0.026	0.650	n.s.
	Vitality (VT)	−0.183	0.001	*p* < 0.01
	Mental health (MH)	0.174	0.002	*p* < 0.01
HRQoL—summary scores	Physical Component Summary (PCS)	0.094	0.101	n.s.
	Mental Component Summary (MCS)	0.066	0.253	n.s.
Health outcomes (NCDs)	Diabetes	0.103	0.073	n.s.
	High blood pressure	0.226	0.000	*p* < 0.001
	High cholesterol	0.156	0.006	*p* < 0.01
	Chronic lung disease	0.203	0.000	*p* < 0.001
	Heart disease	0.068	0.234	n.s.
	Arthritis	0.074	0.199	n.s.
	Kidney disease	0.037	0.520	n.s.
	No disease/none reported	−0.242	0.000	*p* < 0.001

n.s., not significant.

Conversely, residential density exhibited a negative correlation with vitality (*ρ* = −0.183, *P* = 0.001), indicating that individuals in higher-density areas may experience greater fatigue or lower perceived energy levels. No significant associations were found between density and general health (*ρ* = −0.036, *p* = 0.530), social functioning (*ρ* = 0.081, *p* = 0.160), bodily pain (*ρ* = 0.026, *p* = 0.650), or the composite scores physical health summary (PHS) and mental health summary (MHS).

Overall, these findings highlight a nuanced relationship between neighborhood density and quality of life: while denser urban environments appear to support social and emotional dimensions of wellbeing, they may simultaneously impose physical and environmental stressors that are associated with lower vitality and comfort.

**Figure 6:**
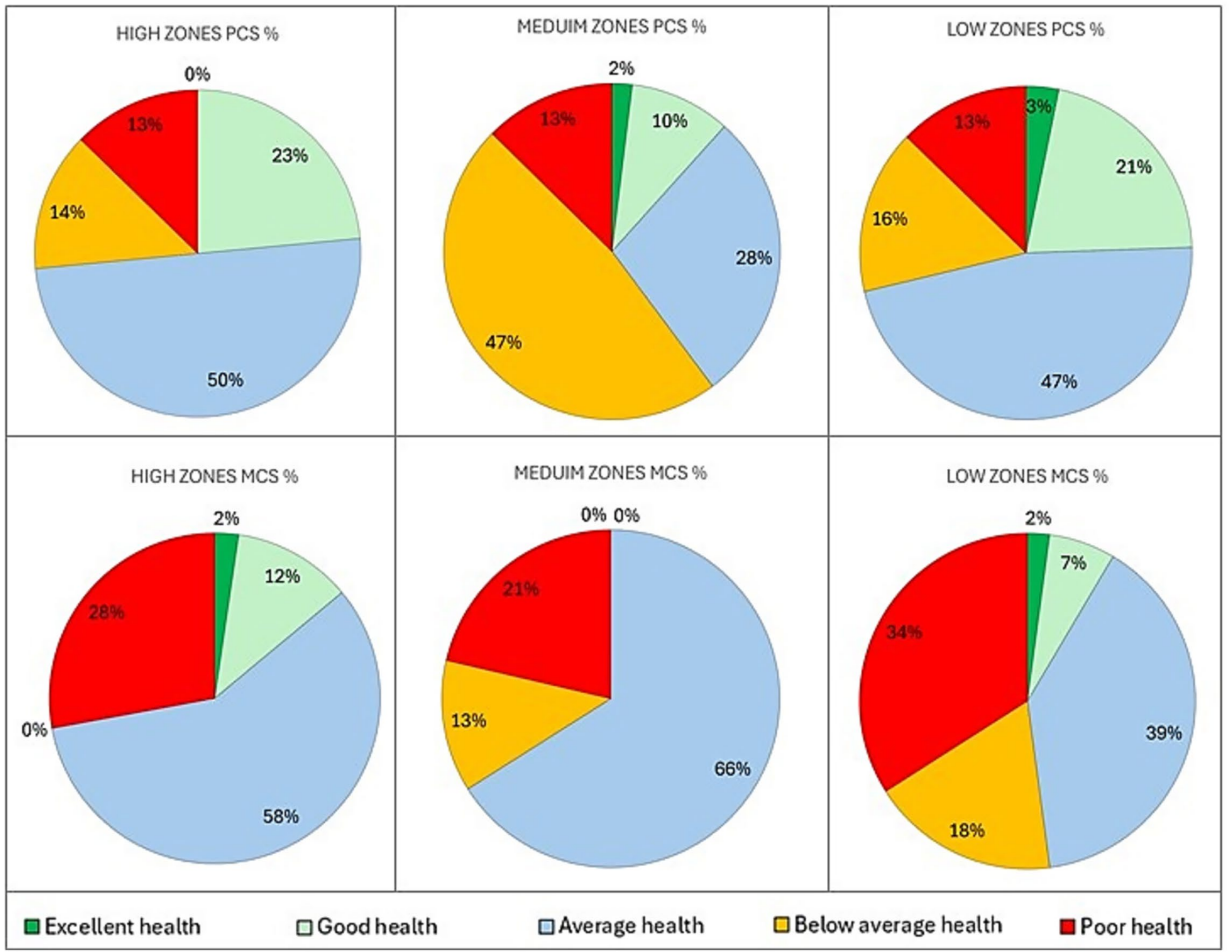
Distribution of Physical Component Summary (PCS) and Mental Component Summary (MCS).

The SF-36 responses were scored and normalized to generate PCS and MCS scores. The PCS results showed variation across density categories: residents in high-density zones reported the highest physical health scores, with 75% RATING their health as ‘Average’ or better, compared with 67% in low-density zones and 36% in medium-density zones (Figure 6).

**Figure 7:**
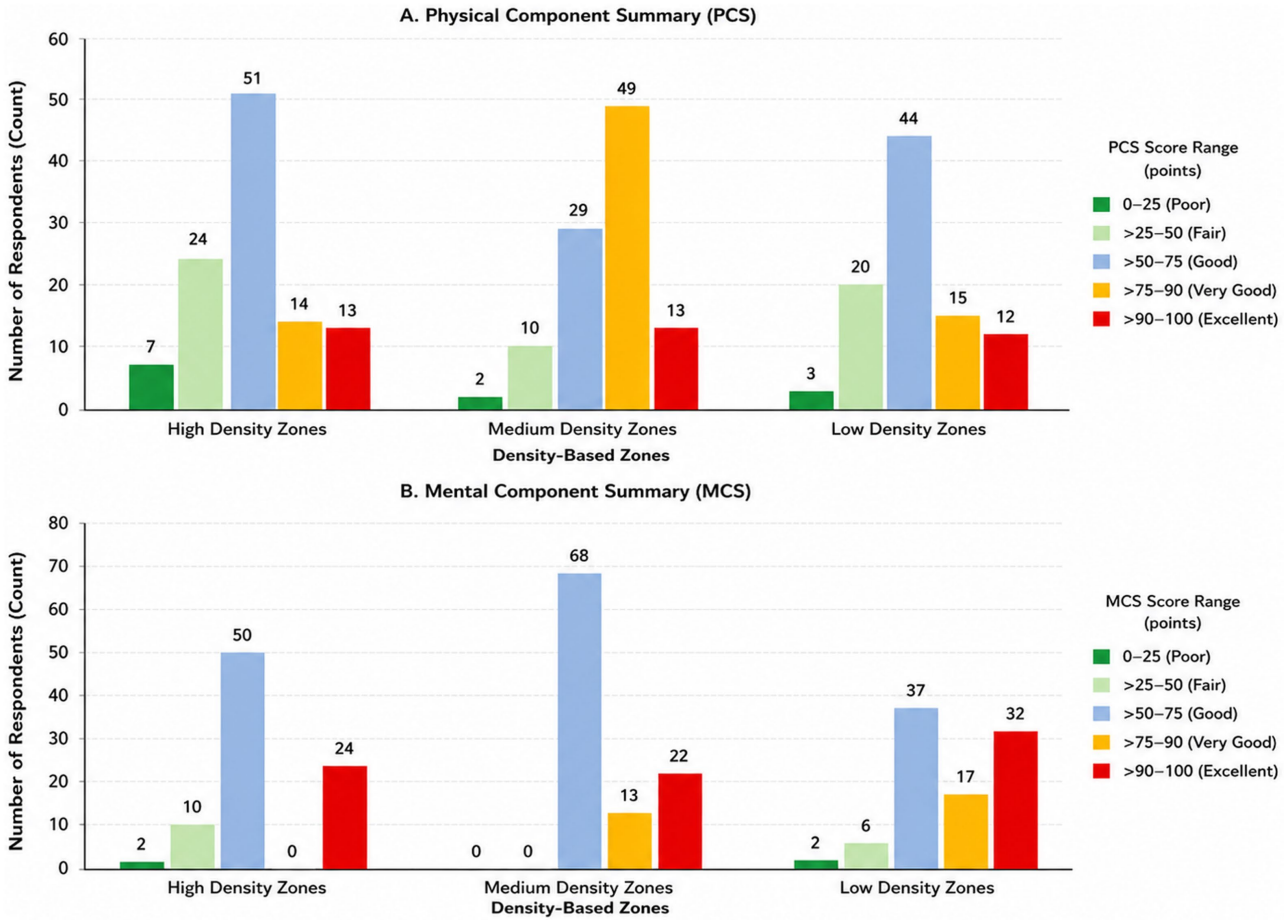
Detailed SF-36 health profile across density-based zones. Distribution of Physical (PCS) and Mental (MCS) Component Summary scores illustrating variations in self-reported health across high-, medium-, and low-density areas.

Furthermore, the impact of density on HRQoL is clearly emphasized (Figure 7). For instance, while physical functioning appears stronger in high-density zones (consistent with continuous PF correlation), domains like Social Functioning and vitality show more participants in lower HRQoL categories in these denser ordinal settings (consistent with negative continuous SF correlation). This domain-specific heterogeneity helps explain why overall summary scores [PCS(T) and MCS(T)] might exhibit complex or non-significant linear trends when density is categorized, as benefits in some domains may be offset by detriments in others.

**Figure 8:**
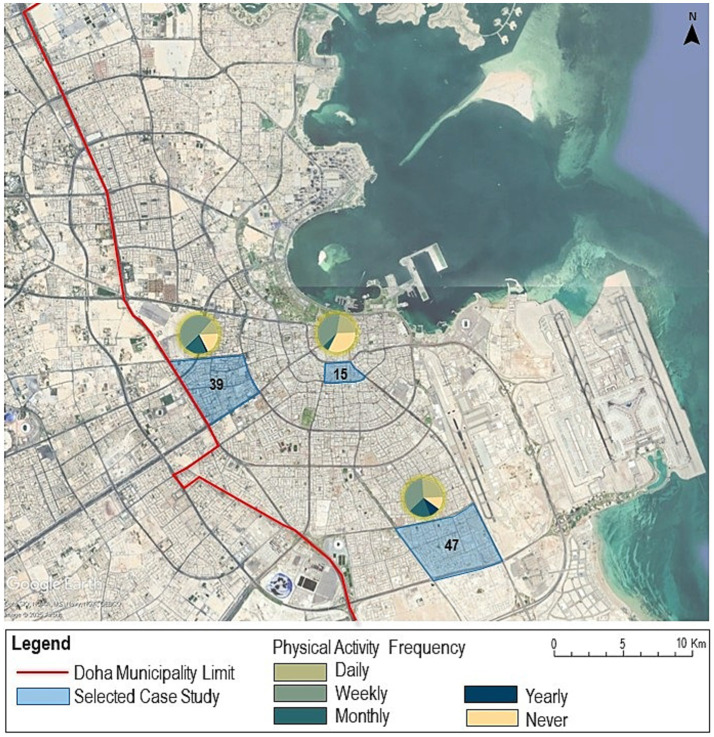
Frequency of physical activity based on questionnaire survey results illustrating variations in activity levels across the studied zones (Image © Google, Data SIO, NOAA, U.S. Navy, NGA, GEBCO, Image © 2025 Airbus).

Residents in high-density areas were significantly more likely to walk for transport (*r* = 0.314, *p* < 0.001). However, perceived environmental quality—not density itself—was the strongest predictor of overall physical activity frequency. Perceptions of a pleasant and encouraging environment for physical activity were strongly correlated with higher activity levels (*r* = 0.262, *p* < 0.001). The spatial variation in reported physical activity frequency is depicted in (Figure 8).

**Figure 9:**
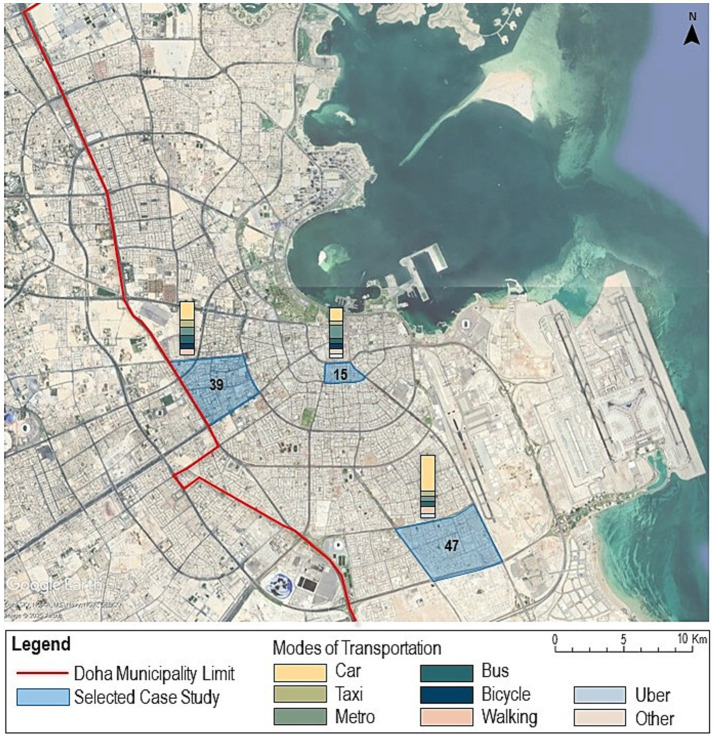
Dominant modes of transportation based on questionnaire survey results illustrating variations in modal share across the studied zones (Image © Google, Data SIO, NOAA, U.S. Navy, NGA, GEBCO, Image © 2025 Airbus).

Chi-square analyses indicated significant associations (all *p* ≤ 0.01) between residential density categories and modes of transport (Figure 9). Higher density favored metro use (46.7% in high density) and walking (31.77%). Car use was most prevalent in low-density areas (92.5%). BMI categories also significantly associated with Uber, metro, and bus use (all *p* ≤ 0.036). A particularly striking finding emerged regarding transport mode and health: walking as a primary mode of transport was positively correlated with heart disease (*r* = 0.105, *p* = 0.011). This is unlikely to be a causal link; rather, it suggests that individuals who walk out of necessity in Doha’s harsh climate may belong to more vulnerable populations exposed to other risk factors like heat stress and air pollution.

#### 4.1.1 Internal consistency of the SF-36 subscales

Internal consistency of the SF-36 subscales was assessed in the present sample using Cronbach’s *α* (Table 4). Reliability was excellent for physical functioning (*α* = 0.94) and good for role physical (*α* = 0.79) and role emotional (*α* = 0.74). General Health (*α* = 0.69) and bodily pain (*α* = 0.67) showed acceptable to marginal reliability, consistent with the small number of items in each subscale.

**Table 4:** Internal consistency (Cronbach’s *α*) of the SF-36 subscales in the present sample.

SF-36 subscale	*k*	Cronbach’s *α*	Standardized *α*	Interpretation
Physical functioning (PF)	10	0.936	0.939	Excellent
Role physical (RP)	4	0.792	0.793	Good
Role emotional (RE)	3	0.740	0.741	Acceptable
General health (GH)	5	0.689	0.716	Acceptable
Bodily pain (BP)	2	0.669	0.671	Marginal
Mental health (MH)	5	0.558	0.0595	Below conventional threshold
Vitality (VT)	4	0.498	0.501	Below conventional threshold
Social functioning (SF)	2	Not calculable in this sample	Not calculable in this sample	Translation artefact (see note)

*k* = number of items. Standardized *α* reported alongside the raw α to account for item-level variance differences. Reverse-coded items were transformed prior to analysis following (39).

In the Arabic-language survey administered in this study, the two Social Functioning items (Q20 and Q32) were found to function as near-identical indicators rather than as two independent angles on the construct, owing to translation conventions in Arabic. Cronbach’s *α* was therefore not meaningfully calculable for this two-item subscale. This is consistent with concerns raised in prior Arabic SF-36 adaptation studies regarding the discriminability of these two items.

Vitality (*α* = 0.50) and Mental Health (*α* = 0.56) showed lower internal consistency than reported in the original Arabic SF-36 validation (46). Diagnostic correlations indicated that this reflects a substantive measurement pattern rather than a coding artefact: positively-worded items (e.g., “full of energy”, “a lot of energy”) clustered together (*r* = 0.59, *p* < 0.001), and negatively-worded items (e.g., “worn out”, “tired”) also clustered together (*r* = 0.49, *p* < 0.001), but the two clusters were essentially independent of each other (*r* ≈ −0.06 to 0.15). A similar pattern is evident in the Mental Health subscale. This positive–negative item separation has been documented in several cross-cultural SF-36 adaptation studies and suggests that, in the present sample, positively and negatively worded affect items may capture related but partially distinct constructs.

Cronbach’s *α* could not be meaningfully calculated for the Social Functioning subscale: the two items (Q20 and Q32) functioned as near-identical indicators in the present sample, owing to convergence in their Arabic translation. This is a known issue in Arabic SF-36 adaptations and is acknowledged in the Limitations section.

In light of these results, the principal inferential analyses use the SF-36 PHS and MHS T-scores rather than individual subscale scores. The summary scores aggregate information across multiple subscales (PHS draws primarily from PF, RP, BP, and GH; MHS draws from VT, SF, RE, and MH), reducing the influence of any single subscale’s lower internal consistency. The lower Vitality and Mental Health α values are nonetheless acknowledged transparently and revisited in the Limitations section.

#### 4.1.2 Adjusted analyses of summary health scores

To examine whether zone-level differences in self-reported health persisted after accounting for socio-demographic characteristics, adjusted general linear models were conducted for the PHS and MHS T-scores, controlling for age, gender, nationality, and employment status (Table 5). Eight respondents were excluded from the adjusted models due to missing data on one or more covariates, yielding a working sample of *N* = 301.

**Table 5:** Adjusted general linear models for Physical (PHS) and Mental (MHS) Health summary scores (*N* = 301).

Source	df	PHS *F*	PHS *p*	PHS *η*^2^*p*	MHS F	MHS p	MHS *η*^2^*p*
Corrected Model	9	7.26	<0.001	0.188	4.81	<0.001	0.133
Zone (Density)	2	5.03	0.007*	0.034	0.13	0.883	0.001
Age	2	10.48	<0.001*	0.069	1.13	0.325	0.008
Gender	1	0.64	0.425	0.002	4.25	0.040*	0.015
Nationality	1	0.001	0.971	0.000	0.003	0.953	0.000
Employment Status	3	3.68	0.013*	0.038	4.90	0.002*	0.049

*Adjusted *R*^2^ = 0.162 (PHS) and 0.105 (MHS).

For PHS, the overall model was significant [*F*(9, 283) = 7.26, *p* < 0.001, adjusted *R*^2^ = 0.162]. Significant adjusted effects were observed for zone [*F*(2, 283) = 5.03, *p* = 0.007, partial *η*^2^ = 0.034], age [*F*(2, 283) = 10.48, *p* < 0.001, partial *η*^2^ = 0.069], and employment status [*F*(3, 283) = 3.68, *p* = 0.013, partial *η*^2^ = 0.038], while gender (*p* = 0.425) and nationality (*p* = 0.971) were not significant. Bonferroni-adjusted pairwise comparisons indicated that residents of the high-density zone (Zone 15) reported significantly higher adjusted PHS scores than residents of the medium-density zone (Zone 39) [mean difference = 3.52, 95% CI (0.79, 6.25), *p* = 0.006]; the low-density zone (Zone 47) did not differ significantly from either of the other two zones (Table 6).

**Table 6:** Adjusted estimated marginal means and Bonferroni-adjusted pairwise comparisons for PHS by zone.

Panel A: estimated marginal means			
Zone	Adjusted mean (PHS)	Std. error	95% CI
Zone 47 (low density)	46.28	0.93	[44.45, 48.11]
Zone 39 (medium density)	43.87	1.05	[41.82, 45.93]
Zone 15 (high density)	47.39	1.07	[45.28, 49.50]

Panel B: pairwise comparisons (Bonferroni-adjusted)				
Comparison	Mean difference	Std. error	*P* (Bonferroni)	95% CI
High (Zone 15)—medium (Zone 39)	**+3.52**	1.14	0.006*	[0.79, 6.25]
High (Zone 15)—low (Zone 47)	+1.11	1.30	1.000	[−2.02, 4.25]
Low (Zone 47)—medium (Zone 39)	+2.41	1.27	0.175	[−0.64, 5.46]

Means are adjusted for age, gender, nationality, and employment status. Asterisk (*) indicates *p* < 0.05. For MHS, no pairwise zone differences reached significance after Bonferroni adjustment (all *p* = 1.000); the corresponding table is omitted.

For MHS, the overall model was also significant [*F*(9, 283) = 4.81, *p* < 0.001, adjusted *R*^2^ = 0.105]. However, the pattern of effects differed markedly from PHS. Zone was no longer associated with mental health after adjustment [*F*(2, 283) = 0.13, *p* = 0.883, partial *η*^2^ = 0.001], and Bonferroni-adjusted pairwise comparisons revealed no significant zone contrasts. Instead, employment status emerged as the strongest predictor [*F*(3, 283) = 4.90, *p* = 0.002, partial *η*^2^ = 0.049], followed by gender [*F*(1, 283) = 4.25, *p* = 0.040, partial *η*^2^ = 0.015]. Age and nationality were not significant predictors of mental health.

Together, these adjusted analyses indicate that the relationship between residential density and self-reported health is domain-specific. Zone-level differences in physical health remain evident after accounting for demographic and socio-economic composition, suggesting an independent association between the built environment and physical functioning. In contrast, the bivariate associations between density and mental health appear to be largely accounted for by the demographic and employment composition of each zone, with individual socio-economic position more closely linked to mental health than residential density per se. These patterns reinforce the broader argument that the relationship between urban form and health is mediated by environmental quality and social context rather than density alone.

### 4.2 Clinical health outcomes verses built environment quality

To validate and contextualize the self-reported survey findings, we analyzed objective clinical NCD prevalence data from PHCCs across the three study zones. The clinical data (Table 7) reveal a modest but notable pattern linking higher residential density with elevated rates of key NCDs, particularly blood pressure and chronic lung diseases. For instance, Zone 15 (high-density) shows comparably high prevalence of both blood pressure (17.2%) and chronic lung disease (5.4%), despite having the smallest registered population. Meanwhile, Zone 39 (medium-density) exhibits the highest absolute burden across all NCD categories due to its much larger population base, though its percentage rates for diabetes (15.1%), blood pressure (19.5%), and chronic lung disease (7.0%) are only slightly higher than those of Zone 15. In Zone 47 (low-density), clinical prevalence remains consistently elevated—especially for blood pressure (19.8%)—suggesting that lower-density suburban living does not necessarily translate into reduced NCD prevalence.

**Table 7:** Clinical non-communicable disease (NCD) prevalence total number and percentage across the three study zones, based on PHCC clinical health records, categorized by residential density (high-, medium-, and low-density areas).

Zone		Clinical health records								
		Diagnosed NCD								
		Total *N*	Diabetes		Blood pressure		Heart disease		Chronic lung	
			*N*	%	*N*	%	*N*	%	*N*	%
15	High-density zone	3,537	563	15.9	607	17.2	202	5.7	191	5.4
39	Medium-density zone	12,913	1,954	15.1	2,522	19.5	711	5.5	908	7.0
47	Low-density zone	8,573	1,370	16.0	1,695	19.8	485	5.7	547	6.4

These patterns indicate that residential density does not exhibit a simple linear or protective relationship with NCD prevalence. Instead, the observed health outcomes reflect a multifactorial density–health dynamic, shaped by lifestyle behaviors, mobility dependence, walkability limitations, exposure to air pollution, and varying access to health and community services. The differing NCD rates across the three zones underscore the importance of triangulating multiple data sources and demonstrate the inherent complexity of interpreting density-related health outcomes in fast-growing, hot-arid Gulf cities.

The Spearman correlation analysis further reinforces the complex relationship between residential density and health outcomes in Doha. As presented in Table 3, several statistically significant positive associations emerged between density and key non-communicable diseases. Higher density was significantly correlated with increased prevalence of high blood pressure (*r* = 0.226, *p* < 0.001), high cholesterol (*r* = 0.156, *p* = 0.006), and chronic lung disease (*r* = 0.203, *p* < 0.001), indicating that residents in denser areas may face greater exposure to environmental or behavioral risk factors that elevate chronic disease burden. A weak positive association was also observed for diabetes (*r* = 0.103), though it did not reach statistical significance (*p* = 0.073), suggesting a trend worth exploring in larger samples. In contrast, the strongest association was found for the absence of any NCD, which showed a significant negative correlation with density (*r* = −0.242, *p* < 0.001). This indicates that residents in higher-density zones were significantly less likely to report being free of chronic disease, supporting the broader pattern of heightened health risks in compact urban settings. Notably, no significant correlations emerged for heart disease, arthritis, kidney disease, or stroke, suggesting that their prevalence may be influenced by additional mediating variables—such as age, lifestyle, or access to healthcare—that extend beyond density alone. Overall, these results highlight that density is not inherently health-promoting in Doha; its effects depend on how well environmental quality and urban infrastructure mitigate stressors linked to NCD risk.

To establish an objective physical context for the health findings, the study conducted a detailed analysis of pedestrian infrastructure quality and land use distribution in the three zones. For the pedestrian infrastructure quality, the analysis reveals a stark divergence in sidewalk quality that generally aligns with density categories (Table 8).

- High-density zone (15): Sidewalk quality is mixed, with approximately 68.37% of the pedestrian network classified as good, reflecting meaningful variation in infrastructure provision across this compact, high-density zone.
- Medium-density zone (39): The pedestrian infrastructure in this zone is of exceptionally high quality, with approximately 96.8% of sidewalks classified as good. Despite this strong physical provision, health outcomes remain mixed, underscoring that infrastructure quality alone is insufficient to ensure health-promoting behavior.
- Low-density zone (47): The pedestrian environment is demonstrably the weakest.

**Table 8:** Quantitative analysis of sidewalk condition across the studied zones.

Zone	Total sidewalk in a good condition (m^2^)	Total sidewalk in a medium condition (m^2^)	Total sidewalk in a bad condition (m^2^)	Percentage of good condition sidewalks %	Percentage of medium condition sidewalks %	Percentage of poor condition sidewalks %
15	1,020	337.5	135	68.37%	22.61%	9.05%
39	797397.5	1368.5	1,234	96.8%	1.70%	1.50%
47	1,260	750	262.5	55.45%	32.99%	11.55%

**Figure 10:**
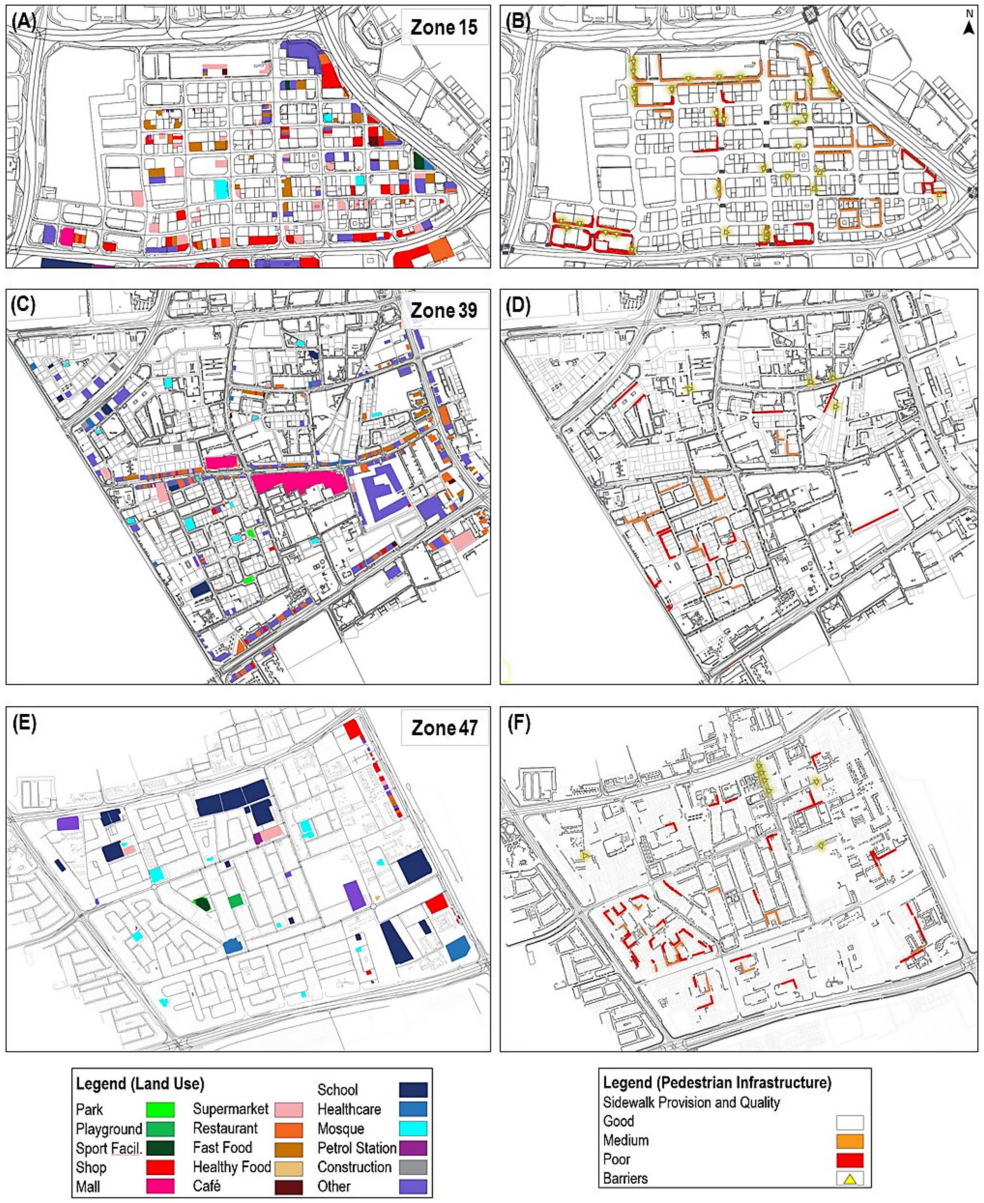
Spatial distribution of land use and pedestrian infrastructure quality across the three study zones. **(A)** Land use distribution in Zone 15 (high-density zone); **(B)** pedestrian infrastructure quality in Zone 15; **(C)** land use distribution in Zone 39 (medium-density zone); **(D)** pedestrian infrastructure quality in Zone 39; **(E)** land use distribution in Zone 47 (low-density zone); and **(F)** pedestrian infrastructure quality in Zone 47. The maps illustrate variations in functional land use patterns and sidewalk conditions across differing residential density contexts.

The built environment profile of Zone 15 reveals the complex interplay between residential density, land use diversity, and pedestrian infrastructure quality as determinants of urban health in Doha. As a high-density zone, the area exhibits a vibrant mix of commercial, institutional, and service-oriented land uses—such as shops, cafés, restaurants, schools, and healthcare facilities—distributed throughout the urban fabric (Figure 10A). This functional mix enhances accessibility and supports local-level walkability, offering opportunities for social interaction and active mobility. However, the benefits of compactness are constrained by the limited presence of parks, playgrounds, and shaded pedestrian routes. Field observations show that while sidewalks are generally available, their quality varies significantly, with many segments obstructed, discontinuous, or lacking thermal comfort features (Figure 10B). The result is a spatial paradox: density fosters proximity and potential walkability, yet deficiencies in environmental quality and pedestrian design reduce its health-promoting value. Exposure to heat, noise, and air pollution, coupled with a scarcity of green and open spaces, further diminishes physical and mental well-being. Overall, the analysis demonstrates that in Zone 15 (and similar high-density areas in Doha) urban health outcomes are shaped not by density alone, but by how effectively land-use diversity and built environment quality are integrated to create a comfortable, safe, and socially inclusive public realm.

On the other hand, the built environment profile of Zone 39, a medium-density urban area, reveals a more balanced but less intense pattern of land-use diversity compared to high-density zones such as Zone 15. The distribution of commercial, institutional, and service functions—including shops, cafés, restaurants, mosques, schools, and healthcare facilities—is concentrated along major streets, providing moderate accessibility and supporting localized walkability (Figure 10C). However, large residential blocks, surface parking, and fragmented pedestrian networks interrupt spatial continuity, limiting the ease and comfort of movement on foot. Field observations show that sidewalks are often uneven, discontinuous, or unshaded, exposing pedestrians to heat stress and discouraging walking during much of the day (Figure 10D). Although a few parks and playgrounds exist, the provision of green and open spaces remains insufficient to serve the resident population or mitigate environmental discomfort. Consequently, while the medium density of Zone 39 promotes proximity and potential social interaction, deficiencies in pedestrian infrastructure and environmental quality constrain its health-promoting capacity. The analysis underscores that in such medium-density settings, the quality of the built environment—particularly shading, greenery, and pedestrian safety—plays a more critical role in shaping urban health outcomes than density itself, highlighting the need for integrated design interventions that enhance comfort, accessibility, and well-being.

Moreover, the built environment profile of Zone 47, classified as a low-density urban area, illustrates a predominantly residential configuration with limited functional diversity. Large housing plots dominate the landscape, while small pockets of community facilities—such as mosques, schools, and healthcare centers—are sparsely distributed. Commercial and service establishments, including cafés, small shops, and food outlets, are scattered along the main arterial roads, offering minimal local accessibility and insufficient support for vibrant street activity (Figure 10E). The spatial arrangement reflects a clear separation between residential and non-residential uses, resulting in long walking distances and weak functional integration. Although a few green spaces and playgrounds exist, they are isolated and poorly connected, offering only localized recreational value. Field observations confirm a strong dependence on private vehicles: discontinuous street networks, wide vehicular roads, and the absence of shaded or continuous sidewalks limit pedestrian comfort and discourage walking (Figure 10F). Overall, the low-density character of Zone 47 restricts land-use efficiency, accessibility, and neighborhood vitality. Despite the presence of essential amenities, their dispersed distribution is associated with lower proximity and walkability. The analysis underscores that in such low-density settings, enhancing street connectivity, shade provision, and clustering of daily-use facilities is essential to improve livability, promote active mobility, and foster a more sustainable and health-supportive urban environment.

### 4.3 Synthesis of results

By triangulating data from resident surveys, clinical records, and objective environmental assessments, a holistic and compelling narrative emerges. The complex dynamics observed in the high-density zone (Zone 15) reflect the multifaceted nature of urban density itself. The higher rates of walking reported by residents are a logical outcome of the compact urban form and mixed land use patterns identified in the land use analysis. However, these same spatial characteristics also contribute to environmental stressors, such as crowding, noise, and limited access to open space; that may offset some of the health benefits of increased walkability. This behavioral pattern is further supported by the quantitative evidence of sidewalk quality, with 68.37% of the pedestrian network classified in good condition (Table 8). However, the simultaneous high prevalence of NCDs reveals an environmental paradox. While proximity encourages walking, the poor environmental comfort—marked by limited shading, traffic exposure, and heat stress— may be associated with potential health gains. These findings substantiate the “necessity-driven walking” hypothesis, where walking occurs out of compulsion rather than choice, often in environments hostile to health.

In contrast, the medium-density zone (Zone 39) shows a different pattern. Despite exceptionally high-quality infrastructure, with over 96% rated as being in good condition (Table 8), health outcomes and HRQoL scores remain uneven. Correlation results indicate that several non-communicable diseases are still present in these neighborhoods, even where the physical environment is well maintained, suggesting that infrastructure quality alone is not necessarily associated with better health outcomes. Residents in denser areas are also less likely to be free from chronic disease, which points to vulnerabilities that extend beyond the built environment’s visual quality. These findings suggest that infrastructure quality may be necessary but not sufficient for supporting active, health-promoting behavior. The urban morphology of these areas, characterized by large block sizes, wider streets, and car-oriented design, may be associated with physical and psychological barriers to walking despite the apparent quality of sidewalks. Although quantitative thermal indices were not measured, field observations of limited shading, together with the higher burden of NCDs in dense zones, indicate that thermal conditions may be an important contextual factor shaping outdoor physical activity.

Finally, the low-density zone (Zone 47) presents the most pronounced case of car dependency and sedentary behavior. The spatial separation of land uses (Table 1) and poor pedestrian infrastructure (only 55.45% in good condition, Table 8) severely limit opportunities for active transport. In such environments, daily mobility relies almost exclusively on private vehicles, structurally embedding sedentary lifestyles and limiting incidental physical activity. The physical disconnection of amenities, combined with infrastructural neglect, compounds the challenge of promoting walkability and healthy living in suburban contexts.

Overall, this multi-source triangulation emphasizes that residential density alone is an insufficient predictor of health outcomes in Doha. The results demonstrate that the quality and design of the built environment—particularly the condition of pedestrian infrastructure, thermal comfort, and land-use integration—act as critical mediating variables. These contextual factors determine whether urban compactness fosters health-promoting behaviors or is associated with higher environmental and physiological stress. Hence, the evidence highlights the urgent need for a shift from density-focused planning towards climate-responsive, health-oriented urban design—a principle further elaborated in the following discussion.

## 5 Discussion

This study reveals a complex, context-dependent relationship between residential density, built environment quality, and health outcomes in Doha. Within the Social Determinants of Health framework, the built environment functions as an intermediate determinant, mediating how broader social and physical conditions are translated into health outcomes. Rather than confirming a linear relationship between compactness and wellbeing, the findings suggest that density operates as a contingent variable whose health effects are shaped by infrastructure quality and demographic composition. Higher residential density in Doha was associated with more walking and greater access to services, yet these benefits were offset by environmental disamenities such as heat exposure, lack of shade, and air pollution. Consequently, environmental quality, rather than density alone, emerges as the more decisive determinant of health outcomes. Density appears to contribute positively to health only when supported by shaded walkways, thermally comfortable microclimates, and safe pedestrian infrastructure. In contrast, dense areas lacking these features may amplify heat stress and physical inactivity, thereby exacerbating the urban burden of non-communicable diseases (NCDs).

A second key finding exposes a paradoxical psychosocial pattern: high-density zones exhibited better mental and functional health scores but poorer social functioning and vitality. This duality reflects what may be termed the “wellbeing paradox of compact urbanism,” where functional accessibility and economic efficiency coexist with social fatigue and weakened community cohesion. Additionally, the correlation between walking for transport and higher incidence of heart disease presents an important equity dimension, revealing that those who walk most in Doha are often the least privileged—laborers or low-income residents without car access whose exposure to heat, pollution, and unsafe infrastructure is associated with higher cardiovascular risks rather than mitigating them (46).

A substantial body of research links residential density to positive health outcomes through increased physical activity and improved access to services, predominantly in Western, temperate urban contexts (1, 2). It is also well established that social capital and perceived trust influence the relationship between built form and mental health (3, 4). More recent literature has argued that the functional quality and micro-design of public space, rather than density per se, are associated with the health experience of residents (29, 47). However, these insights have been generated largely from temperate, pedestrian-oriented urban settings, leaving the applicability of density–health pathways in hot-arid, car-dependent cities largely untested.

This study makes three contributions to the urban health and public health literature. First, it provides empirical evidence that the density–health relationship operates differently in hot-arid, car-dependent cities, where climatic and infrastructural deficits can reverse the expected health benefits of compactness. The presence of sidewalks or proximity to amenities does not guarantee active mobility if environmental conditions make walking unsafe or unpleasant—transforming density from a quantitative measure into a qualitative condition.

Second, the study identifies a wellbeing paradox within compact urbanism: functional accessibility may enhance independence and mental stimulation, but it cannot substitute for the social interactions and sense of belonging fostered by human-scaled, inclusive public spaces. In the Gulf context—where outdoor sociality is constrained by climate—designing indoor or semi-outdoor communal spaces becomes an essential public health strategy.

Third, the finding that transport-related walking correlates with higher cardiovascular risk reframes walkability from a behavioral choice to a social necessity, introducing the dimension of exposure equity. This expands the concept of “active mobility” by demonstrating that walkability interventions must move beyond spatial accessibility and address micro-environmental safety and comfort—shade continuity, air quality, and traffic separation. These findings collectively highlight the urgent need for a policy shift from pursuing densification as a quantitative goal toward prioritizing high-quality, climate-responsive urban design in rapidly developing hot-arid cities.

## 6 Limitations of this study

The principal strength of this paper lies in its multi-source, triangulated methodology, which integrates objective and environmental data to capture a holistic view of urban health. By combining clinical NCD prevalence with resident perceptions and built-environment audits, the study bridges epidemiological and spatial approaches, offering a rare cross-disciplinary model for the region. Moreover, the focus on a hot-arid, car-oriented city contributes novel evidence to global debates that have historically centered on temperate, high-income contexts.

Nevertheless, several limitations should be acknowledged. First, the cross-sectional design precludes causal inference and supports only associations, not directionality. Reverse causality cannot be ruled out: while the present analysis proceeds from the theoretical position that built environment features shape NCD outcomes, it is equally possible that zone-level population sorting processes—whereby households with elevated health risk locate in particular residential zones for reasons unrelated to built environment quality—could produce the observed associations. The cross-sectional design does not permit any conclusions about cause and effect, and this limitation should be foregrounded in any policy application of these findings. Second, although triangulation helps reduce single-source bias, the reliance on self-reported data introduces potential recall and perception bias.

Third, the convenience sampling approach, while appropriate for an exploratory design, may limit representativeness across income groups and nationalities. This is a particularly salient concern in Doha, where citizenship status, cultural background, and labor-market position are unevenly distributed across residential zones: Zone 15 is disproportionately comprised of non-citizen labor migrants, whereas Zone 47 has a substantially higher proportion of Qatari nationals. These citizenship-related compositional differences may independently shape health behaviors, healthcare access, and self-reported health perceptions in ways that cannot be fully disentangled from density effects in the present design. Future research should employ stratified sampling with adequate representation of both citizen and non-citizen groups within each zone to enhance comparability and external validity.

Fifth, internal consistency of the SF-36 subscales was re-estimated in the present sample. While most subscales showed acceptable to excellent reliability, the Vitality and Mental Health subscales showed lower internal consistency than reported in the original Arabic SF-36 validation. Diagnostic correlations indicated that positively-worded and negatively-worded affect items formed two related but partially independent clusters in the present sample, a pattern previously documented in several cross-cultural SF-36 adaptation studies. To mitigate the impact of this issue on the principal analyses, the GLMs use the SF-36 Physical and Mental Health Summary scores, which aggregate across multiple subscales. Nonetheless, MHS-based findings should be interpreted with this measurement limitation in mind. In addition, the two Social Functioning items were found to function as near-identical indicators in their Arabic-translated form, precluding meaningful estimation of internal consistency for this subscale. Future Arabic-language administrations of the SF-36 in Qatar and the broader Gulf region would benefit from cognitive interviewing to refine the wording of Q20 and Q32 so that they capture distinguishable dimensions of social functioning as intended in the original instrument. Direct household income was not measured in the survey; employment status was therefore used as a proxy for socio-economic position in the adjusted models. While employment status is a recognized correlate of economic activity and income, it is an imperfect substitute and may not fully capture variation in household economic circumstances. Future research should incorporate direct measures of income, household composition, and physical activity to permit more granular adjustment.

## 7 Conclusion

This paper challenges the prevailing assumption that urban density is inherently health-promoting. Evidence from Doha shows that density is associated with a context-dependent influence; enhancing accessibility and mental well-being while simultaneously heightening exposure to environmental stressors such as heat, pollution, and limited green space. Thus, the health implications of density are not universal but mediated by the quality and design of the built environment. Density supports well-being only when environmental and social conditions mitigate its potential risks.

The study demonstrates that environmental quality—rather than density alone—is the decisive determinant of health outcomes. While compact areas encourage walking and access to services, these benefits are undermined by thermal discomfort and unsafe pedestrian conditions, emphasizing the need for a shift from quantitative densification to qualitative, resilience-oriented urban design. A second key finding reveals a psychosocial paradox: high-density zones exhibit better mental health but weaker social functioning, suggesting that accessibility cannot replace the sense of belonging fostered by inclusive public spaces. In hot-arid climates like Doha, semi-outdoor, shaded, and human-scaled spaces are essential to sustain social well-being.

Theoretically, the study reframes density as a qualitative condition shaped by environmental, social, and behavioral resilience. It positions urban form as a structural determinant of health, aligning urban planning with the WHO Health in All Policies agenda.

Ultimately, quantitative densification alone cannot achieve well-being goals. Sustainable cities require qualitative transformation—anchored in high-quality urban design, equitable mobility systems, and social cohesion. This approach, consistent with Qatar National Vision 2030, offers a pathway for Gulf cities to move beyond growth metrics toward urban environments that actively promote health, equity, and resilience.

## 8 Implications for practice and advancement of research

The findings have important implications for urban policy and planning in Doha and other hot-arid, rapidly urbanizing contexts. Municipal Planning Authorities should treat built-environment design as a public health policy trigger, not merely an architectural concern. In particular, health impact assessments (HIAs) could be integrated into zoning permits for high-density districts, with minimum requirements for shaded walkability, pedestrian continuity, and thermally safe public space. Rather than simply calling for “more shade,” policy should specify enforceable standards for climate-responsive walkability, including a measurable Shaded Walkability Index for high-density developments. Infrastructure investment should therefore prioritize not only connectivity, but also heat protection, safety, and accessibility, since these conditions are more closely aligned with mobility and health outcomes in hot-arid settings.

As density rises, socially sustainable urban forms become essential. Inclusive public spaces, community hubs, and interconnected green networks can strengthen cohesion, mitigate stress, and promote mental health. Embedding Health Impact Assessments (HIA) into planning—linking environmental and health indicators—aligns with Qatar’s National Health Strategy (2018–2022) and Vision 2030, positioning urban design as a proactive determinant of public health.

The study also advances theoretical understanding of urban well-being as an interdisciplinary construct, demonstrating how environmental quality, design, and social equity jointly determine health outcomes. Building on these findings, future research should pursue:

1. Multivariate and spatial models to isolate the effects of built-environment variables (e.g., shading, land-use mix, amenity proximity) on health.
2. Longitudinal and quasi-experimental studies evaluating interventions such as shaded walkways or park redevelopments.
3. Qualitative and participatory methods to explore “necessity-driven walking” and lived experiences of heat, safety, and accessibility among marginalized groups.
4. Comparative studies across Gulf and MENA cities to test the model’s adaptability and policy relevance.

In summary, this research offers a conceptual and empirical foundation for a new urban health paradigm: viewing density not as a numeric target but as a dynamic condition shaped by climate, design, and equity. It provides a replicable framework for achieving healthier, more sustainable, and resilient cities across the Global South.

## Data Availability

The data analyzed in this study is subject to the following licenses/restrictions: Available upon request. Requests to access these datasets should be directed to hameda.janahi@qu.edu.qa.
